# Diagnostic utility of LEF1 and β-catenin in WNT pathway tumors with CTNNB1 mutation

**DOI:** 10.1186/s12957-025-03675-8

**Published:** 2025-01-29

**Authors:** Can Li, Lingdan Dong, Li Zhu, Wenbin Guan

**Affiliations:** 1 Department of Pathology, Xinhua Hospital Affiliated to Shanghai Jiaotong University School of Medicine, 1665 Kongjiang Road, Yangpu District, Shanghai, 200092 China; 2 Department of Pathology, Maternal and Child health Hospital of Hubei Province, Wuhan, China

**Keywords:** LEF1, Β-catenin, WNT signaling pathway tumors, CTNNB1 gene mutation, Immunohistochemical marker

## Abstract

**Objective:**

This study aimed to compare the expression of lymphoid enhancer factor 1 (LEF1) and β-catenin in basal cell adenoma (BA), desmoid-type fibromatosis (DF), and pancreatic solid pseudopapillary neoplasm (SPN) to evaluate their diagnostic utility in tumors associated with the WNT/β-catenin signaling pathway harboring the mutation of CTNNB1 gene 3 exon.

**Methods:**

Eighty tumor patients, including 26 BAs, 30 DFs, and 24 SPNs, were analyzed. Immunohistochemical staining was identified positive (nuclear staining of LEF1 and β-catenin in > 50% of tumor cells). The diagnostic rate of LEF1 alone, β-catenin alone, and their combination were compared for each tumor type and all patients.

**Results:**

Compared to β-catenin, when LEF1 alone was used for diagnosis, the diagnostic rate increased by 46.16% for BA, 16.67% for SPN, and 11.25% for all patients, but decreased by 23.34% for DF. The combined use of β-catenin and LEF1 significantly increased the diagnostic ratio in BA (46.16%), SPN (16.67%), and all patients (21.25%), but only marginally in DF (3.33%). In terms of all WNT pathway tumors with CTNNB1 gene mutation encompassed by our study, statistical analysis revealed no significant difference between LEF1 alone and β-catenin alone. However, their combined application was highly significant (*P* = 0.001) .

**Conclusion:**

While β-catenin is commonly used as a marker for WNT pathway tumors, its variable expression and localization can be challenging for diagnosis. Our study emphasizes the importance of LEF1 as a complementary marker to β-catenin in diagnosing BA, DF, SPN, and other WNT pathway tumors activated by exon 3 CTNNB1 gene mutation. The combined use of LEF1 and β-catenin enhances diagnostic accuracy and may help the identification of these tumor types.

## Introduction

WNT-activated tumors commonly harbor the mutation in exon 3 of CTNNB1 gene [1, 2]. β-catenin, a downstream effector of the WNT signaling pathway, undergoes phosphorylation by GSK-3β in the cytoplasm, which is a crucial regulatory step under normal conditions. Phosphorylated β-catenin translocates to the cell nucleus, where it interacts with other proteins to modulate gene expression before undergoing degradation by the ubiquitin-proteasome system [2]. Mutation of CTNNB1 in exon 3 activates the WNT signaling pathway, leading to the accumulation of β-catenin in the nucleus. Consequently, β-catenin nuclear staining is generally regarded as an indicator of WNT-activated tumors.

Despite its significance, the sensitivity of β-catenin varies, and its localization can fluctuate between nuclear/cytoplasm or membrane with background staining. Factors such as antibody clone and uncertain cut-off value further contribute to variability, posing challenges in achieving an accurate diagnosis. Lymphoid Enhancer Factor 1 (LEF1) is a transcription factor that specifically interacts with β-catenin, forming a transcriptional complex in the cell nucleus. This complex regulates the WNT signaling pathway and controls the expression of downstream target genes involved in cell cycle regulation, including c-Myc and Cyclin D1 [3–6].

In this study, we focused on three common WNT-activated tumors, basal cell adenoma (BA), desmoid-type fibromatosis (DF), and pancreatic solid pseudopapillary neoplasm (SPN) to systematically evaluate the expression patterns of β-catenin and LEF1 and analyze their diagnostic value. Compared to β-catenin, LEF1 clear nuclear staining was more easily identified. However, the sensitivity of LEF1, particularly in DF, may be lower than that of β-catenin. The CTNNB1 gene testing couldn’t be conducted under restricted laboratory conditions, moreover, not all cases harbor the gene mutation. Hence, immunohistochemical staining is an economical and rapid approach. Now our study presented that the combined application of LEF1 and the traditional marker β-catenin could screen out β-catenin staining was lacking or in the membrane or cytoplasm. The accuracy and timeliness of pathological diagnosis are very important for the subsequent treatment of patients and for indicating prognosis. Therefore, it is helpful combining use of LEF1 and β-catenin which could select WNT pathway tumors quickly and precisely. Nevertheless, the small sample size, along with the deficiencies in genetic testing, has presented new direction for our future study.

## Materials and methods

### Patient selection

A total of 80 formalin-fixed paraffin-embedded (FFPE) WNT-activated tumors were collected from the Department of Pathology, Xinhua Hospital affiliated with Shanghai Jiaotong University School of Medicine. Clinical data, morphological characteristic, and immunohistochemical results of 26 BAs, 30 DFs, and 24 SPNs were comprehensively analyzed. These 80 cases we selected were all based on the latest WHO diagnostic criteria, with typical morphology features. Only after an accurate diagnosis was confirmed were patients included in our study. We analyzed the expression of β-catenin and LEF1. In this study, one patient with DF originated from the nasal cavity, one with DF located on the back, one with DF located on the left chest wall, two with DF located on the buttocks, and the remaining 25 with DF located on the abdominal wall. All the Hematoxylin and eosin (HE) and immunohistochemically stained slides were reviewed separately by two experienced pathologists and reached a consistent conclusion.

### Immunohistochemistry staining

Immunohistochemistry was performed on 4 μm FFPE tumor tissue sections using monoclonal antibodies against β-catenin (clone 14/Beta-Catenin, 1:200 dilution; BD Transduction Laboratories, San Jose, CA) and LEF1 (clone EP-310, ready to use; ZSGB-BIO, Beijing, China). Staining was performed by incubating the slides with 3,3’-diaminobenzidine (DAB), followed by counterstaining with hematoxylin. Subsequently, the slides were dehydrated using gradients of ethanol and xylene. The immunohistochemistry was performed by an automated stainer.

To date, there is no universally acknowledged positive cut-off value. We have selected a critical threshold of 50% based on the conclusions drawn from previous studies, which may indicate the strongest association with the CTNNB1 gene. For our study, the background staining of β-catenin was uniformly strong. LEF1 staining in DF and SPN was well-distributed with no significant difference. However, LEF1 in BA showed distinctive peripheral myoepithelium expression, a phenomenon noted in previous reports and our study, which we believe aids in BA diagnosis. Therefore, in this study, patients were considered positive when more than 50% of tumor cells exhibited nuclear staining for both β-catenin and LEF1.

Samples from 10 patients of adenoid cystic carcinoma, 5 patients of superficial fibromatosis, and 10 patients of pancreatic acinar cell carcinoma were used as negative control for LEF1 and β-catenin.

### Statistical analyses

Statistical analyses were performed using SPSS for Windows version 19.0 (SPSS Inc., Chicago, IL, USA). A comparison of LEF1 and β-catenin expression was performed using Pearson’s chi-square and Fisher’s exact tests. Statistical significance was set at *p* < 0.05.

## Results

**Fig. 1:**
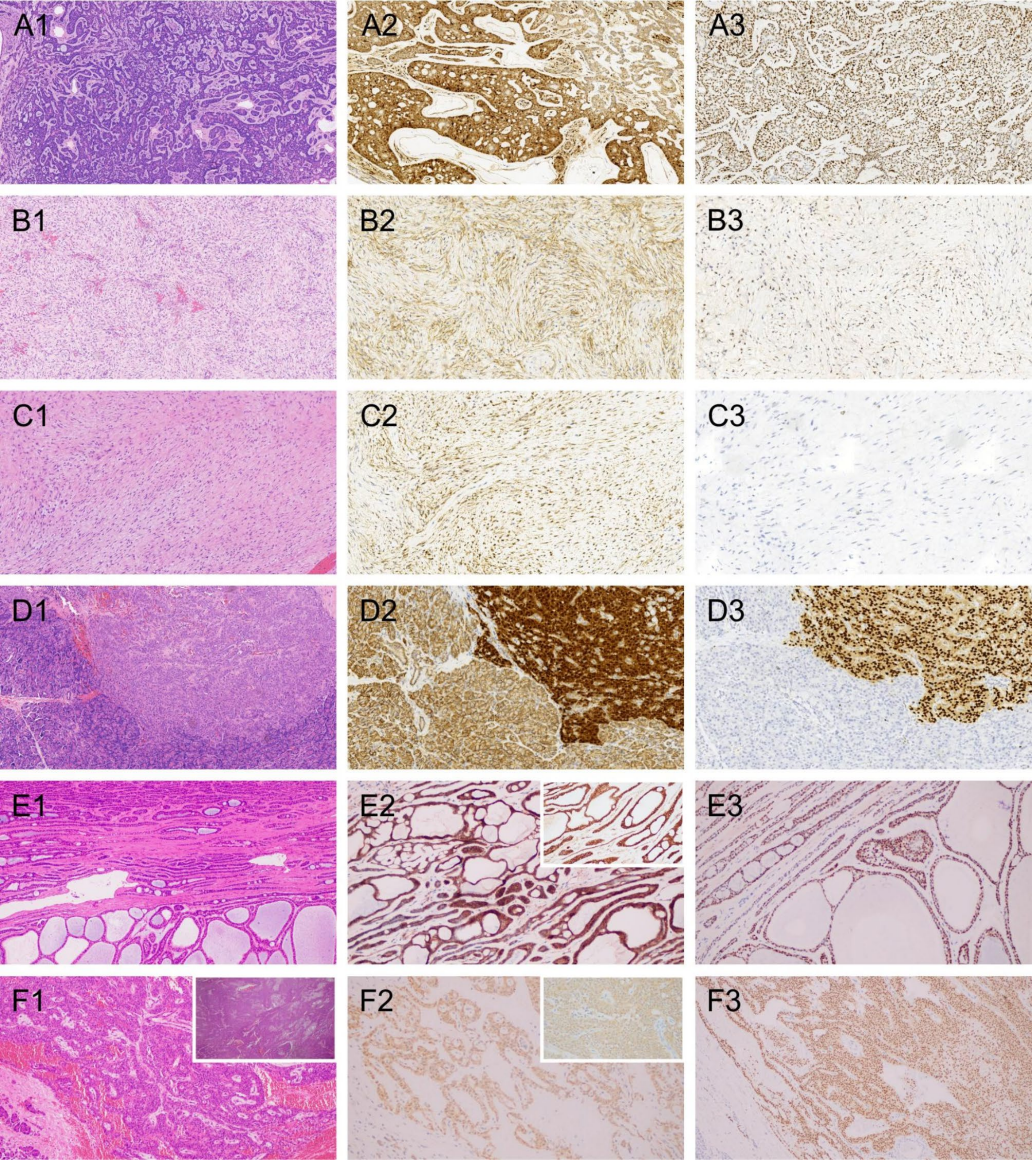
**A** Morphology of BA (A1, H&E staining, original magnification ×100). Tumor cells exhibited focal membranous and nuclear/cytoplasmic positivity for β-catenin (A2, ×200). LEF1 expression was characterized by nuclear staining, mainly located in peripheral myoepithelial cells, with weaker staining in central cell nests (A3, ×200). **B** Morphology of DF originating from the nasal cavity (B1, H&E, original magnification ×100). β-catenin immunostaining revealed a cytoplasmic dotted-like pattern. (B2, ×200). Tumor cells were positive for LEF1 immunostaining (B3, ×200). **C** Morphology of DF (C1, H&E, original magnification ×100). Tumors showed strong and diffuse β-catenin nuclear/cytoplasmic labeling (C2, ×200). LEF1 immunostaining was negative (C3, ×200). **D** Morphology of SPN (D1, H&E, original magnification ×100). Tumors showed a diffuse β-catenin nuclear pattern along with cytoplasmic staining (D2, ×200). LEF1 nuclear staining showed no immunoreaction for normal pancreatic tissue (D3, ×200). **E** Morphology of BA (E1, H&E, original magnification ×100). Tumors showed a β-catenin membranous pattern (E2, ×200; The upper right corner showed magnification ×400). LEF1 nuclear staining was identified (E3, ×200). **F** Morphology of SPN (F1, H&E, original magnification ×100; The upper right corner showed magnification ×400). Tumors showed a β-catenin membranous pattern (F2, ×200; The upper right corner showed magnification ×400). LEF1 nuclear staining was observed (F3, ×200)

The 80 patients with WNT-activated tumor consisted of 26 BAs, 30 DFs, and 24 SPNs. In BA, 10/26 (38.46%) were β-catenin positive, while LEF1 was positive in 22/26 (84.62%) (*p* = 0.001). When combined use of β-catenin and LEF1, 22/26 patients with BA (84.62%) were identified. Among β-catenin expression cases, six exhibited nuclear/cytoplasmic staining, and the other four harbored nuclear/membranous labeling. β-catenin negative group showed membranous or focal cytoplasmic expression, with two patients showing no staining. Accentuated β-catenin staining in the peripheral myoepithelium was noted (Fig. 1A). The scale of β-catenin expression in BA was variable, ranging from focal to diffuse, posing challenges in interpretation. Of the 22 patients with LEF1 nuclear expression, 16 were primarily located in peripheral myoepithelial cells with a lack of or weaker staining in central cell nests. The remaining six cases exhibited diffuse nuclear staining throughout the tumors. In the LEF1-negative expression group, four patients all showing LEF1 staining in 10–30% of tumor cells, with no β-catenin immunoreaction or focal β-catenin membranous staining, suggesting that focal LEF1 expression may have diagnostic significance for BA. Regardless of the proportion, clear nuclear LEF1 labeling was observed in all patients with BA. Our conclusion indicated that whether LEF1 was used alone or in combination with β-catenin, the positive diagnostic rate of BA can be significantly enhanced.

In the patients with DF, β-catenin expression was observed in 26/30 (86.67%). LEF1 was evident in 19/30 (63.33%) (*p* = 0.07). All 26 DFs with nuclear β-catenin expression simultaneously exhibited cytoplasmic staining. A DF case originating from the nasal cavity showed the β-catenin cytoplasmic dotted-like pattern without any nuclear staining, which might routinely be considered negative. However, this patient exhibited LEF1 expression (Fig. 1B). Among the 11 patients with DF classified as LEF1-negative, seven showed 8–10% nuclear expression. The other four patients were completely non-immunoreactive. Moreover, LEF1 staining of endothelial cells within blood vessels served as a positive control to confirm that LEF1 was truely negative. However, strong and diffuse β-catenin nuclear/cytoplasmic labeling was seen in these four patients with LEF1-negative DF (Fig. 1C). For our study, we had a larger number of DF cases presented with weaker or even negative LEF1 staining, and the LEF1 sensitivity for DF diagnosis was somewhat lower. Furthermore, the expression of LEF1 exhibited focal nuclear staining (i.e., the “hot area”). Therefore, both LEF1 and β-catenin are recommended for use in the diagnosis of DF. In this study, the combined use of β-catenin and LEF1 produced a correct diagnosis in 27 DF cases increasing the expression ratio to 90%.

Concerning SPN, β-catenin expression was observed in 20/24 samples (83.33%). LEF1 was detected in 24/24 (100%) cases (*P* = 0.11). All 20 β-catenin expression cases showed a diffuse β-catenin nuclear pattern, along with cytoplasmic staining. (Fig. 1D). Four patients classified as β-catenin negative expression varied in their percentages of membranous expression. However, all were accompanied by diffuse LEF1 nuclear staining. All SPNs demonstrated diffuse and strong nuclear LEF1 labeling. The absence of LEF1 immunoreactivity in normal pancreatic tissue served as a useful marker for evaluating SPN margin.

LEF1 was identified in 65/80 (81.25%) WNT pathway tumors, and β-catenin expression observed in 56/80 (70%). However, an accurate selection of 73/80 (91.25%) of WNT-activated tumors occurred when both β-catenin and LEF1 were simultaneously applied (*p* = 0.001; Tables 1 and 2). For this study, there was no significant difference in DFs and SPNs, which might be attributed to the limited sample. However, LEF1 nuclear staining makes the interpretation easily. Therefore, it still holds considerable practical value. This study marks the initial exploration of their combined diagnostic value. All 80 patients were identified when the cut-off value for LEF1 was not set.

**Table 1:** Comparison of diagnostic results between using LEF1 alone and β-catenin alone

Tumor	Nuclear β-Catenin		LEF1		*p*-value
	Yes	No	Yes	No	
BA (*n* = 26)	10	16	22	4	0.001
DF (*n* = 30)	26	4	19	11	0.07
SPN (*n* = 24)	20	4	24	0	0.11
Total (*n* = 80)	56	24	65	15	0.14

**Table 2:** Diagnostic results of combined use of β-Catenin and LEF1

Tumor	Combined use of β-Catenin and LEF1		Nuclear β-Catenin		*p*-value
	Yes	No	Yes	No	
BA (*n* = 26)	22	4	10	16	0.001
DF (*n* = 30)	27	3	26	4	1
SPN (*n* = 24)	24	0	20	4	0.11
Total (*n* = 80)	73	7	56	24	0.001

## Discussion

A diverse range of WNT-activated tumors have been recognized, such as BA, DF, SPN, hepatocellular adenoma, deep penetrating nevi, medulloblastoma, and endometrial carcinomas. The primary cause of abnormal WNT pathway activation is typically associated with the CTNNB1 gene mutation encoding the β-catenin protein. However, alterations in the APC and AXIN1 have been implicated. The phosphorylation site located in exon 3 of the CTNNB1 gene is a prominent mutation hotspot [7, 8].

Due to morphological diagnostic difficulties, immunostaining is often used to identify these lesions, especially in needle aspiration specimens. While β-catenin is a traditional marker for diagnosing WNT pathway tumors, its evaluation remains challenging. Previous studies exploring the relationship between β-catenin expression and CTNNB1 gene mutation in various WNT-activated tumors have yielded diverse conclusions. The prevailing view once was that only nuclear β-catenin staining was significant. However, recent studies have indicated its inconsistencies. For example, Kim et al. reported eight patients with endometrial carcinoma exhibiting β-catenin cytoplasmic labeling despite CTNNB1 gene mutation [9]. Explanations for the lack of strict association between the CTNNB1 gene mutation and β-catenin nuclear staining have been provided in the literature. Hagen et al. reported that membranous accumulation of β-catenin can activate WNT pathway transcription [10]. In the current study, a few patients with BA and SPN showed exclusive membranous β-catenin staining. (Figure 1E and F) Another study by Tirbulo et al. suggested the WNT signaling pathway could be independent of the nuclear aggregation of β-catenin protein [11]. Kafri proposed that the rate of change in the nuclear accumulation of β-catenin, rather than the total quantity, correlated better with the Wnt pathway [12]. The variable pattern of β-catenin, while posing challenges in evaluation, holds unique value. For instance, cytoplasmic expression may indicate a worse prognosis in renal carcinoma [13]. However, for the majority of WNT pathway tumors, nuclear β-catenin staining almost invariably predicts CTNNB1 gene mutation. A definitive cut-off value remains elusive, and different conclusions exist. Fattet et al. suggested that extensive and nuclear β-catenin labeling is necessary for CTNNB1 gene mutation, while Wang et al. opined that even a minimal percentage of the β-catenin nuclear pattern should be considered significant [2, 14]. β-catenin exhibited diffuse and strong staining in almost all patients with DF, indicative of a relatively narrower spectrum of CTNNB1 mutation in DF.

β-catenin, acting as a cadherin-binding protein, plays a crucial role in cell adhesion. However, upon forming a complex with members of the T-cell factor/LEF family of proteins in the cell nucleus, it transforms into a transcriptional activation factor [15]. While it has been recognized that LEF1 tends to associate with β-catenin in the nucleus to exert its function, instances where LEF1 acts independently to activate the WNT pathway have been observed [16, 17].

In this study, a lower β-catenin expression ratio was observed in BA compared to LEF1. Similar to β-catenin, accentuated LEF1 staining was observed in myoepithelial cells surrounding the tumor nests, with clear nuclear expression. Patients categorized as negative for β-catenin displayed cytoplasmic or membranous staining in approximately 20–40% of tumor cells, with two patients showing no β-catenin immunoreactivity yet exhibiting approximately 30% LEF1 nuclear expression. Approximately 15% of adenoid cystic carcinomas and pleomorphic adenomas may display β-catenin nuclear staining [18, 19]. LEF1 is expressed in other salivary gland tumors, but its expression in BA is significantly higher [16, 20]. Our research demonstrated that all BAs harbored different ranges of LEF1 nuclear staining. So LEF1 has a distinct advantage in the diagnosis of BA.

The mutation in CTNNB1 (at codon 41 or 45 in exon 3) or APC can lead to the activation or dysregulation of the WNT pathway, which is the main pathogenic mechanism of DF [21]. The translocation of β-catenin protein from the cytoplasm to the nucleus due to CTNNB1 gene mutation makes the β-catenin nuclear pattern meaningful [22]. The relationship between DF, β-catenin nuclear staining, and CTNNB1 gene mutation is intricate, and we cannot simply assume that nuclear β-catenin positivity equals CTNNB1 gene mutation. Yamada et al. reported two patients without β-catenin nuclear staining that had CTNNB1 gene mutation with a distinct cytoplasmic “dotted” pattern [21]. β-catenin cytoplasmic staining was associated with DF, mainly related to the 41 A CTNNB1 mutation [22–25]. In this study, there was a fortunate coincidence that a patient with DF that occurred in the nasal cavity exhibited unique dotted ring staining. Koike et al. described that β-catenin nuclear-negative DFs harbored CTNNB1 mutation, while the mutation could not be identified in nuclear β-catenin expression cases [22]. A relevant explanation has been provided, indicating that S45 phosphorylation is the initial process of β-catenin degradation and that the S45F mutation may prevent the first step of phosphorylation. The subsequent degree of phosphorylation may vary depending on the specific codon of the mutation, leading to different staining patterns of β-catenin in different patients [26]. Additionally, Yamada et al. confirmed that different clones of β-catenin have diagnostic differences in DF, and clone β-catenin 14 had low specificity for the diagnosis of DF [21]. Lower expression of β-catenin is associated with the CTNNB1 mutation in codon 45 (45 F) and is more prone to recurrence [21]. Researchers proposed that high nuclear β-catenin staining was associated with higher 5-year survival [27]. Goto et al. reported that, using clone β-catenin 14 antibodies with a 10% cut-off value, patients with scar often exhibited β-catenin nuclear staining [28]. Other soft tissue tumors and scar tissues may harbor CTNNB1 mutations or exhibit nuclear β-catenin expression [27]. Ng et al. reported varying degrees of β-catenin nuclear staining in various tumors, such as solitary fibrous tumors, endometrial stromal sarcomas, synovial sarcomas, fibrosarcomas, and clear cell sarcomas [29]. Amary et al. reported that approximately 72% of fibromatous lesions exhibited nuclear β-catenin expression [30]. While using the marker β-catenin may seem challenging due to various interfering factors and low specificity, it possesses advantages. Previous studies showed that the sensitivity of LEF1 in the diagnosis of DF was lower than that of β-catenin, and the specificity of LEF1 was poor. Zou et al. revealed that 14 patients with scar showed LEF1 expression, but only one patient showed weak positivity for β-catenin [31]. However, only applying the marker β-catenin is not perfect; in this study, one patient with DF occurring in the nasal cavity, a relatively rare location, showed that the diagnostic performance of β-catenin was inferior to that of LEF1. The case exhibited a focal β-catenin dotted-like cytoplasmic pattern, while LEF1 showed strong nuclear staining. The diagnosis of spindle cell tumors in soft tissues is difficult, and no perfect markers are available. Despite the difficulties, the CTNNB1 gene mutation still provides high specificity for the diagnosis of DF, and the nuclear β-catenin pattern has suggestive value. However, the expression of the β-catenin protein is not stable, and for patients with β-catenin negative, the joint use of LEF1 can improve the diagnostic rate of DF.

Our study demonstrated β-catenin nuclear and cytoplasmic staining in SPN, consistent with previous reports, with a few patients showing pure nuclear expression [32, 33]. Certain patients exhibited membrane staining. However, LEF1 in SPN showed diffuse nuclear labeling, and unlike β-catenin, which harbored diffuse membranous staining in normal pancreatic tissues, LEF1 was negative. The positive rate of β-catenin and LEF1 in SPN were similar and were indiscriminate with known conclusions [34, 35]. Other pancreatic tumors, such as pancreatic neuroendocrine tumors, ductal adenocarcinoma, and pancreatoblastomas, may exhibit β-catenin staining; still, they mainly show focal nuclear/faint cytoplasmic staining or membranous labeling, and pancreatoblastomas present β-catenin staining mainly confined to squamoid corpuscles. Additionally, 25% of acinar cell carcinoma exhibit alteration of the β-catenin signaling pathway, resulting in diffuse β-catenin cytoplasmic/nuclear staining [36–38]. LEF1 expression has been described in other pancreatic tumors, but the conclusions are not consistent, possibly due to the limitations in sample capacity, antibody selection, and cut-off value. For example, Singhi et al. demonstrated that LEF1 is negative in acinar cell carcinomas and ductal adenocarcinomas, whereas McHugh et al. reported a weak-to-moderate LEF1 nuclear pattern in pancreatic acinar cell carcinomas and ductal adenocarcinomas [33, 34]. LEF1 expression in pancreatic neuroendocrine tumors is almost always negative; however, occasionally, patient with positive expression have been reported [33, 36]. LEF1 expression in pancreatoblastomas is mainly confined to squamoid corpuscles [32, 33, 36]. Therefore, conclusions from the related literature showed that LEF1 and β-catenin have slightly lower specificity in the diagnosis of SPN; however, they still have value due to their diffuse expression pattern differing from other pancreatic tumors. In our study, four patients identified as β-catenin negative expression in SPN showed diffuse and strong LEF1 nuclear staining. Thus, in comparison with β-catenin, LEF1 has higher sensitivity, harbors clean, clear staining, and is easy to evaluate the tumor border, making it a better marker for diagnosing SPN. However, LEF1 was positively stained in normal T and pro-B lymphocytes, which should be considered in needle biopsy specimens.

To date, there have been few studies on the expression of β-catenin and LEF1 in WNT pathway tumors. In this study, we shared limited 80 tumor cases, including 26 BAs, 30 DFs and 24 SPNs. Combined use of β-catenin and LEF1 significantly increased the diagnostic ratio in BA, DF, SPN and all 80 cases by 46.16%, 3.33%, 16.67% and 21.25%, respectively. Only using LEF1, there was no statistically significant difference (*P* = 0.14). However, the combined application of β-catenin and LEF1 in diagnosing the 80 WNT pathway tumors showed a distinct significance (*P* = 0.001). Our perspective is relatively novel, and the goal was not to correlate the expression of LEF1 and β-catenin proteins with CTNNB1 gene mutation. Although molecular technology is advancing, morphology and immunohistochemistry remain the basis and core of pathological diagnosis. We used a simple and economical method to screen for these three rare WNT pathway tumors, which can quickly exclude morphologically similar tumors and make an accurate diagnosis. This is particularly important for patients with limited financial capacities or hospitals that have not yet implemented the corresponding molecular technologies. Meanwhile, our findings may have positive implications for the diagnosis of other related WNT signaling pathway tumors. However, our study was limited by insufficient tumor types to demonstrate the diagnostic performance of the combined use of LEF1 and β-catenin in WNT-activated tumors. It is well-known that the smaller sample is accompanied by relatively large random errors, resulting in insufficient statistical significance. So we hope that more data could be available. Meanwhile, the diagnostic value of β-catenin and LEF1 in other WNT pathway tumors merits exploration. For instance, the CTNNB1 gene mutation can result in 90% WNT-activated medulloblastoma, which can induce alterations in the amino acid residues at the phosphorylation site of β-catenin, and LEF1 can form a transcriptional complex with β-catenin in the nucleus. Consequently, LEF1 is also an excellent auxiliary marker for diagnosing WNT-activated medulloblastoma. Multi-institutional collaboration, the increased number cases, and conducting CTNNB1 gene testing can render the conclusion more convincing.

## Data Availability

No datasets were generated or analysed during the current study.
